# Evaluation of Automated Alert and Activation of Medical Emergency Team in Head and Neck Cancer Patients Using Early Warning Score at Tertiary Level Hospital in North India

**DOI:** 10.7759/cureus.31428

**Published:** 2022-11-12

**Authors:** Mohib Ahmed, Fuzail Sarwer, Gunjan ., Moazzam Jawaid, Sakshi Raina, Abdullah Alnazeh

**Affiliations:** 1 Department of Critical Care and Anaesthesiology, Raj Hospitals, Ranchi, IND; 2 Department of Oral Medicine, Diagnosis and Radiology, Sarjug Dental College and Hospital, Darbhanga, IND; 3 Department of Orthodontics, Dental Officer Ex-Servicemen Contributory Health Scheme (ECHS) Polyclinic, Samastipur, IND; 4 Department of Orthodontics, King Khalid University, Abha, SAU

**Keywords:** patients, head and neck cancer, activation system, automated alert, met

## Abstract

Background: Improved patient safety for those undergoing treatment of head and neck cancers depends on prompt identification of warning indicators from severely critical patients and appropriate treatment. As a result, many hospitals all around the world have implemented quick response techniques, including medical emergency teams (MET), which have systems and personnel that are highly trained to deal with patients who are deteriorating. Nowadays, automated activation and alert programs are also being discussed.

Aim: To compare the patient outcomes in two conditions: one before the application of the automated MET alert and activation program and the other after the application of the automated MET alert and an activation program was used.

Methods and Materials: There was an examination of clinical data on MET-managed patients before and after the computerized alert and activation approach was put in place. The study comprised all adult head and neck cancer patients who were treated by the MET between March 1, 2017, and December 31, 2021. The physiologic abnormalities of the patient at the moment of the MET initiation were recorded as causes for MET activation when the MET was alerted by the computerized alert, and activation system. From activation through deactivation, the MET intervention lasted. Hospital mortality served as the study's primary outcome. The duration of stay at the hospital and unscheduled ICU hospitalizations were secondary outcomes. Medical records from hospitals were examined retrospectively to get information on clinical outcomes.

Results: The percentage of unplanned admissions in ICU was greater in the pre-implementation stage (22.34%) as compared to that in the post-implementation stage (13.56%) (p value<0.001). The duration of stay of patients at the hospital also got reduced in the post-implementation phase (13.23 ±1.47 days) as compared to the pre-implementation stage (24,76± 1.12 days) (p<0.001). The median time of derangement of activation of MET was greater in the pre-implementation stage (62 minutes) as compared to the post-implementation stage (34 minutes) (p-value <0.001). The most common complications leading to MET activation in a pre-implementation phase were neurological and respiratory complications. On the other hand, overall deterioration was the most common cause of MET activation. The mortality rate of patients in the pre-implementation stage was 36.23% as compared to 22.12% in the post-implementation stage (p<0.001).

Conclusion: The hospital experienced improved clinical outcomes with the adoption of an automated alarm and activation system using a cumulative weighted scoring methodology, which significantly reduced the time from disruption to MET activation.

## Introduction

Head and neck cancer patients in hospital wards frequently display atypical physiological symptoms hours before complications take place [1]. Improved patient safety for those undergoing treatment of head and neck cancers depends on prompt identification of warning signs from severely critical patients and appropriate treatment. As a result, many hospitals all around the world have implemented quick response techniques, including medical emergency teams (MET), which have systems and personnel that are highly trained to deal with patients who are deteriorating [2]. However, earlier research [3,4] offered debatable support for the benefits of MET. Weak areas in the quick system, such as lower sensitivity for identifying patient deterioration and lags in MET activation, are one cause of differences in the results [5]. Recognizing patients who are critically ill or in danger of deterioration is the first step in activating the MET [6]. It is advised that all patients admitted to an acute care hospital should be screened using physiological surveillance and trigger systems [7]. A computerized information system is an advantageous component of monitoring systems because it can increase the reliability and accuracy of deterioration detection [8]. Computerized MET activation could reduce activation delays compared to the conventional method, which is initiated by phone calls [9-12]. As a result, we predicted that implementing a computerized MET alert and activation system would enhance MET's early activation for patients who were clinically worsening and lead to better patient outcomes. Limited information is known, nevertheless, regarding how automated activation systems affect clinical outcomes in non-selected hospitalized patients in critical care, as well as the time it takes from the deterioration of a patient up to MET activation. To learn more about how MET activation affects head and neck cancer patients, we compared how they did in two different situations: before and after the automated MET alert and activation program were used. 

## Materials and methods

After the MET program at Raj Hospitals in Ranchi, Jharkhand, was launched, all head and neck cancer patients who received MET care were prospectively registered. We examined clinical data on MET-managed patients before and after the computerized alert and activation approach was put in place to answer the core research objective of whether the application of the computerized alert and activation approach for MET is connected with health outcomes in head and neck cancer patients admitted to the critical care and anesthesiology departments and then transferred to the general ward. Because this study is an observational one, the institutional reviewing board approved it and waived the need for informed consent with IRB number IEC/2016/1245. Before analysis, patient data was also de-identified and anonymized.

Study population

The study comprised all adult head and neck cancer patients who were managed in the general ward by the MET between March 1, 2017, and December 31, 2021. A prior study [13] contained some clinical data for individuals treated between 2013 and 2018. Patients from areas other than the main ward, such as the outpatient department (OPD) or day care unit, were not included in the study because the active screening program with a computerized alert and activation mechanism was only deployed in the general ward. Additionally, MET requests for which there was no outcome data were disregarded. When patients were seen by the MET more than once during the same hospital stay, the first time was considered a baseline MET activation.

Those patients with a Modified Early Warning Score (MEWS) of seven or above at the moment of MET activation were considered for the main study to address the major research objective of analyzing the impact of the computerized alert and activation approach with the MEWS [14]. The final patient population was separated into patients belonging to the pre-implementation phase and patients belonging to the post-implementation phase. Previous articles [13,15,16] have given details about the functioning of the hospital's MET system. The MET is made up of committed critical care physicians and surgeons who provide 24-hour coverage. They include intensive care associates and attending physicians. Before the automation system was put into place, doctors and nurses would directly call the MET at a designated phone number when a patient fulfilled any one of the conditions listed above. Additionally, activation was permitted despite the lack of physiological abnormalities that fulfil the criteria when the medical staff was worried about alterations in the medical problems of their patient. An automatic alert and activation mechanism were added to the initial MET activation procedure. During the trial period, requests for MET engagement were offered to all patients, regardless of their do-not-resuscitate status. If the MET is activated, it is anticipated that it will arrive within 10 minutes, finish patient evaluations in 30 minutes, and order diagnostic testing and therapeutic interventions that are pertinent to the patient's condition. After evaluation and treatment plans are given by the MET, patients who need therapy and observation that can't be done anywhere else are sent to the ICU with complications such as emergency organ failures, stroke, and bleeding disorders. Patients whose conditions are stable stay in the hospital's general wards.

Implementation of the automated alert and activation system

The computerized alert and activation approach based on the MEWS was used by the MET to launch an active screening program across all ward patients. When nurses entered the patient's vital indicators into the electronic health care record, the MEWS was generated automatically, employing five physiological signals (systolic blood pressure (SBP), heart rate, respiration rate, body temperature, and degree of awareness). An automated alarm was delivered to members of MET in the form of a text in actual time, 24 hours a day, seven days a week, whenever the MEW score was seven or greater, and the Medical Emergency Team engaged automatically. Patients who agreed to a do-not-resuscitate command were omitted from the active screening, and the hospital's digital medical record was used to capture information on the patient's code status. While MEWS was color-coded in the patient profile in the computerized medical record system, alerts were not shown to the ward's doctors and nurses. As a result, the ward's medical personnel could identify the patient's MEWS status: green color code for MEWS 0 to 2, yellow color code for MEWS 3 and 4, orange color code for MEWS 5 and 6, and red color code for MEWS 7 or higher. When feasible, vital signs of the patient were captured at the bedside quickly following examination using only a laptop or transportable device. Without a set hospital policy, the number of vital sign measurements was determined by the attending physician. However, vital signs were often recorded at least four times each day and sometimes more frequently if the patient's clinical condition changed. Every time a patient's vital signs were recorded for the first time, MEWS was updated right away.

Data collection and clinical outcomes

As quickly as feasible after the event, a MET member entered the required information into a registry for all MET activations brought on by calls and automated alerts. Patient demographic characteristics, the method that activated the MET (call or automated alert), the causes of the MET activation, the time of the first substantiated physiological disorder, MEWS, the times the MET was activated and deactivated, the vital signs at those times, the interventions the MET provided, and the patient's state following the clinical episode were all collected and recorded. This data was added the next day to check hospital medical records before registering for registration data quality control. The physiologic abnormalities of the patient at the moment of the MET initiation were recorded as causes for MET activation when the MET was alerted by the computerized alert and activation system. From activation through deactivation, the MET intervention lasted. Hospital mortality served as the study's primary outcome. The duration of stay at the hospital and unscheduled ICU hospitalizations were secondary outcomes. Medical records from hospitals were examined retrospectively to get information on clinical outcomes. Because self-imposed limits on treatment could affect the clinical outcomes, patients who cut back on therapy after MET intervention were not included in the analyses of outcomes.

Statistical analysis

To compare the traits and clinical results between the two stages before and after the computerized alert and activation program was put in place, descriptive statistics were used. A Mann-Whitney U test was used to examine the median values and interquartile ranges of continuous variables. Where appropriate, chi-squared tests, as well as Fisher's exact tests, were used to assess categorical variables, which were reported with numbers and percentages. The odds ratios of active screening using the computerized alert and activation methods were calculated using the logistic regression approach, and the risk variables for hospital mortality in patients in general wards who had clinical deterioration were identified. The OR (odds ratio) of every predictor with 95% confidence intervals was used to represent the results (CIs). For all analyses, a 2-tailed P-value of equal or below 0.05 was regarded as statistically significant. The statistical program SPSS Inc. SPSS for Windows, Version 14.0. Chicago, SPSS Inc. was used to examining the data (IBN, USA).

## Results

**Figure 1:**
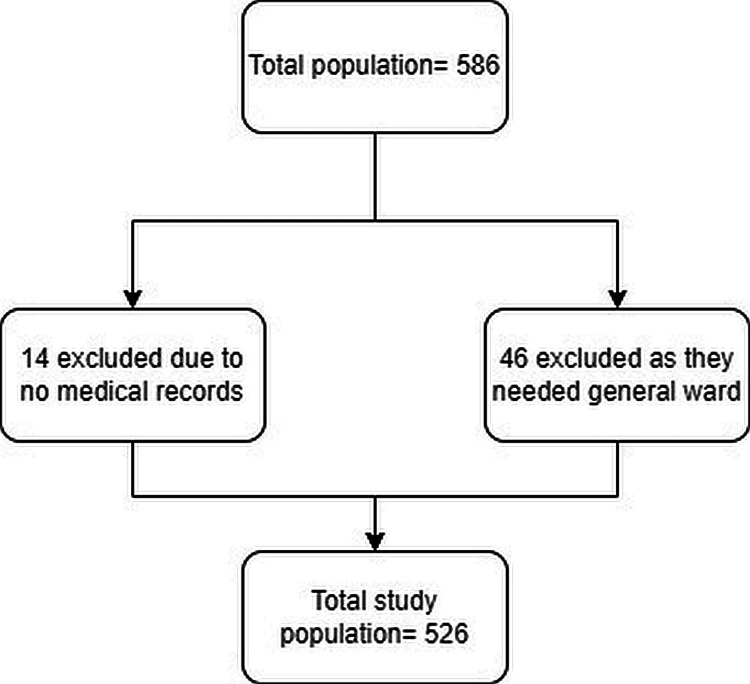
Flow chart for population included

In this study, the total number of patients with head and neck cancer managed by MET was 586. Among them, 46 patients were out of the general ward, and the medical records of the other 14 patients were able to be retrieved. Therefore, they were excluded from the study (Figure 1).

Finally, 526 patients managed by MET were found eligible for the study. The number of study participants before the pre-implementation of the computerized alert and activation method for the engagement of MET was 261; the number of study participants after the pre-implementation of the computerized alert and activation method for the engagement of MET was 265. The mean age of study participants in the pre-implementation group was 62.43 years, while the mean age of study participants in the post-implementation group was 62.7 years. Males outnumbered females in both groups (78.6 and 80.1%). Most of the patients in both groups were from the medical department (79.9% and 78.2%).

The percentage of unplanned admissions in the ICU was greater in the pre-implementation stage (22.34%) as compared to that in the post-implementation stage (13.56%) (p-value 0.001). Patients' hospital stays were also reduced in the post-implementation phase (13.23 days) compared to the pre-implementation stage (24.76 days) (p = 0.001). The median time of derangement of activation of MET was greater in the pre-implementation stage (62 minutes) as compared to the post-implementation stage (34 minutes) (p-value = 0.001). The most common complications leading to MET activation in the pre-implementation phase were neurological and respiratory complications. On the other hand, overall deterioration was the most common cause of MET activation. The mortality rate of patients in the pre-implementation stage was 36.23% as compared to 22.12% in the post-implementation stage (p = 0.001) (Table 1).

**Table 1:** Comparison of details of head and neck cancer patients before and after implementation of automated MET system ICU: Intensive care unit MET: Medical emergency teams

	Age (years)	Male (%)	Unplanned ICU admissions	Stay at hospital (days)	Median time from derangement of MET activation	MET calls meeting criteria for activation	Mortality
Pre-implementation (n=261)	62.43±2.36	78.6	22.34%	24,76± 1.12	62 minutes	Neurological and respiratory	36.23%
Post-implementation (n=265)	62.79± 1.25	80.1	13.56%	13.23 ±1.47	34 minutes	Overall deterioration	22.12%
P value	0.427	0.518	<0.001	<0.001	<0.001	<0.002	<0.001

In the multivariate evaluation, stage of cancer (III & IV), cardiopulmonary pulmonary resuscitation, High-flow nasal cannula (HFNC) and noninvasive ventilation (NIV), respiratory system as a cause for MET activation, and delay in the time of activation of MET were significantly related to an elevated risk of patient mortality, while implementation of the automated activation and alert system of MET and medical and surgical therapy given by MET was significantly related to reduced risk of patient mortality (Tables 2-5).

**Table 2:** General predictors of MET calls for mortality of patients in hospital MET: Medical emergency teams

	Implementation of automated alert system	Age>70	Stage of head and neck cancer	MET activation during weekend	MET activation during nighttime hours	Time from derangement to MET activation, 10 min
Adjusted OR	0.84	1.27	1.51	1.18	1.12	1.02
95% CI	0.57–0.96	0.97–1.66	1.04–2.17	0.93–1.55	0.79–1.27	1.11–1.03
P-value	0.016	0.114	0.023	0.195	0.671	0.002

**Table 3:** Reason for MET activation as predictors for mortality of patients in hospital

	Respiratory system	Circulatory system	Bedside concern
Adjusted OR	1.46	1.16	0.65
95% CI	1.10–1.93	0.84–1.58	0.32–1.30
P-value	0.009	0.366	0.220

**Table 4:** Vital sign at the time of MET activation as predictors of mortality in hospital

	Hypotension	Tachycardia (Heart rate>110 beats/min)	Tachypnea (Respiratory rate>20 breath/min)	Fever (Temperature>38.3)
Adjusted OR	1.25	0.96	0.96	0.45
95% CI	0.94–1.64	0.64–1.45	0.67–1.46	0.35–0.59
P-value	0.149	0.786	0.827	<0.002

**Table 5:** MET intervention as predictors of mortality in hospitals

	HFNC/NIV	Airway management	Cardiopulmonary resuscitation	Bolus fuid administration	Medication therapy	Advice or consultation only
Adjusted OR	1.79	1.27	5.16	0.59	0.75	0.98
95% CI	1.08–2.99	0.88–1.85	2.03–13.11	0.44–0.79	0.57–0.97	0.71–1.35
P-value	0.028	0.215	0.001	<0.001	0.027	0.836

## Discussion

According to the authors' knowledge, this is the first study to evaluate the role of automated activation and alert systems for MET in head and neck cancer patients at a tertiary-level hospital of Raj Hospitals in Ranchi. In our study, patients were divided into two categories: One category included patients managed by MET before the application of the automated activation and alert system for MET, and the other included patients managed by MET after the application of the automated activation and alert system for MET. It was found that the percentage of unplanned admissions in the ICU was greater in the pre-implementation stage as compared to the post-implementation stage. The duration of stay of patients at hospitals was also reduced in the post-implementation phase as compared to the pre-implementation stage. The median time of derangement of activation of MET was greater in the pre-implementation stage as compared to the post-implementation stage. The most common complications leading to MET activation in the pre-implementation phase were neurological and respiratory complications. On the other hand, overall deterioration was the most common cause of MET activation.

A reduced proportion of ICU admissions is associated with automated stimulation of the MET employing electronic medical record-based screening criteria [9], although the influence of automated stimulation on fatality has not been detected in the study. The period from dysregulation to MET activation was decreased by automated activation, suggesting quicker activation. In a different study, the use of automated alarm or notification systems based on predetermined physiologic parameters was also linked to a decrease in standard hospital mortality. However, the impact of reducing the time to the engagement of MET on outcomes was not obvious [13-15]. Another automated quick-response team activation that was put in place in general medicine departments and used an institution-specific prediction model was linked to a decrease in complications, deaths, and cardiopulmonary arrest from one year to the next [11].

The use of a computerized prediction model to identify high-risk subjects for whom therapies by rapid-response teams are linked with decreased mortality was recently discovered by major multicenter cohort research [12], although the influence on time to action was not documented. Therefore, we looked at whether an automated alert and activation strategy based on the MEWS score could reduce the amount of time it took for a patient to become deranged before their MET was activated and whether this would result in a meaningful improvement in their clinical outcomes. Our study shows that, following the implementation of the computerized alert and activation mechanism for MET in head and neck cancer patients, clinical outcomes, such as unexpected ICU admission, inpatient death, and duration of hospitalization improved [16-19].

Objective evaluations have been suggested to enhance the early detection of unexpected declines. The most common grading method is an overall weighted approach based on variations in vital signs. In this investigation, the MEWS, which was calculated and used automatically, was used to turn on the MET automatically [20-22]. During the period prior to implementation, criteria made up exclusively of single aberrant physiologic variables with predetermined cut-offs were utilized as MET activation triggers. This single-parameter methodology had the benefit of being easy to use and consistent, but it had limitations because it was unable to detect small changes in a number of physiologic parameters simultaneously. Cumulative weighted point systems, like the MEWS, on the other hand, can track clinical progress and find big changes in patients' vital signs [14,22].

Additionally, compared to single factors, aggregate scoring methods are more accurate at identifying the risk of unfavorable outcomes [23]. To help the medical staff in this study identify overall alterations in the patient's vital signs, color-coded MEWS was shown in the patient profile in the electronic health record interface. And even in the patient subgroup with MEWS of less than seven, the percentage of patients who triggered MET during the post-application period but did not satisfy a single physiologic condition for MET notifications as per the non-automated method was higher. So, the use of MEWS made it easier to find patients who were getting worse but didn't meet the requirements for a single metric but did have problems with several physiologic variables.

But calculating cumulative weighted scoring systems manually is difficult and susceptible to inaccurate measurements [24]. As a result, while allowing for potential errors, computerized computation and categorization of the aggregate score system enhance the identification of patients who are susceptible to unfavorable outcomes. An abnormal number can cause MET activation. This procedure, however, still necessitates physician activation of the MET, limiting its automated usefulness. As soon as our alarm system identified patients with clinical deterioration, MET was instantly activated. Although patient impairment was readily identified, delays in MET activation were induced by several cultural barriers to care escalation or MET interaction [25]. In particular, nurses at the bedside frequently delay calling the MET [25].

Although both doctors and nurses were permitted to contact the MET at our center, in reality, doctors made practically all of the MET calls. This shows that when aberrant vital signs were discovered, nurses first informed the junior doctor of the patient's clinical state. After examining the patient, the resident doctor called the MET. The interval between deterioration and MET activation was greatly reduced since the automatic alarm and activation system avoids this procedure. In many clinical settings, it has been shown that acting quickly when a patient is very sick is helpful, and shortening the time between deterioration and MET activation helps skilled medical professionals evaluate and treat the patient sooner.

The study had some limitations. First, the study's inherent retrospective observational design posed limitations. Even though all MET participants were taught how to record each variable, some information may have been missed, and recollection bias may have affected how accurate the results are.

## Conclusions

The hospital experienced improved clinical outcomes with the adoption of an automated alarm and activation system using a cumulative weighted scoring methodology, which significantly reduced the time from disruption to MET activation. If clinical impairment is found, automatic alarm and activation software make sure that the MET is turned on quickly. This helps avoid delays that could happen with a system that isn't automated. 
